# Supporting Accurate Interpretation of Self-Administered Medical Test Results for Mobile Health: Assessment of Design, Demographics, and Health Condition

**DOI:** 10.2196/humanfactors.8620

**Published:** 2018-02-28

**Authors:** Jess C Hohenstein, Eric PS Baumer, Lindsay Reynolds, Elizabeth L Murnane, Dakota O'Dell, Seoho Lee, Shion Guha, Yu Qi, Erin Rieger, Geri Gay

**Affiliations:** ^1^ Department of Information Science Cornell University Ithaca, NY United States; ^2^ Department of Computer Science and Engineering Lehigh University Bethlehem, PA United States; ^3^ School of Applied and Engineering Physics Cornell University Ithaca, NY United States; ^4^ Sibley School of Mechanical and Aerospace Engineering Cornell University Ithaca, NY United States; ^5^ Department of Mathematics, Statistics, and Computer Science Marquette University Milwaukee, WI United States; ^6^ Department of Chemistry Rice University Houston, TX United States

**Keywords:** mobile health, health informatics, patient-generated health data, user-computer interface, decision making, patient-centered care

## Abstract

**Background:**

Technological advances in personal informatics allow people to track their own health in a variety of ways, representing a dramatic change in individuals’ control of their own wellness. However, research regarding patient interpretation of traditional medical tests highlights the risks in making complex medical data available to a general audience.

**Objective:**

This study aimed to explore how people interpret medical test results, examined in the context of a mobile blood testing system developed to enable self-care and health management.

**Methods:**

In a preliminary investigation and main study, we presented 27 and 303 adults, respectively, with hypothetical results from several blood tests via one of the several mobile interface designs: a number representing the raw measurement of the tested biomarker, natural language text indicating whether the biomarker’s level was low or high, or a one-dimensional chart illustrating this level along a low-healthy axis. We measured respondents’ correctness in evaluating these results and their confidence in their interpretations. Participants also told us about any follow-up actions they would take based on the result and how they envisioned, generally, using our proposed personal health system.

**Results:**

We find that a majority of participants (242/328, 73.8%) were accurate in their interpretations of their diagnostic results. However, 135 of 328 participants (41.1%) expressed uncertainty and confusion about their ability to correctly interpret these results. We also find that demographics and interface design can impact interpretation accuracy, including false confidence, which we define as a respondent having above average confidence despite interpreting a result inaccurately. Specifically, participants who saw a natural language design were the least likely (421.47 times, *P*=.02) to exhibit false confidence, and women who saw a graph design were less likely (8.67 times, *P*=.04) to have false confidence. On the other hand, false confidence was more likely among participants who self-identified as Asian (25.30 times, *P*=.02), white (13.99 times, *P*=.01), and Hispanic (6.19 times, *P*=.04). Finally, with the natural language design, participants who were more educated were, for each one-unit increase in education level, more likely (3.06 times, *P*=.02) to have false confidence.

**Conclusions:**

Our findings illustrate both promises and challenges of interpreting medical data outside of a clinical setting and suggest instances where personal informatics may be inappropriate. In surfacing these tensions, we outline concrete interface design strategies that are more sensitive to users’ capabilities and conditions.

## Introduction

**Background**

With the increasing pervasiveness of self-monitoring technology, much of the health data that had previously been gathered and analyzed by experienced practitioners are now being collected and interpreted by individuals outside of traditional health care settings [1]. The widespread use of personal tools for collecting, analyzing, and providing feedback about health data poses broad questions regarding how people make sense of this information. What kinds of medical data are appropriate to self-monitor? Without relevant training and practice, can laypersons accurately interpret their own health measures? Furthermore, are people confident in their ability to take control of their own health in these ways, without consultation with a health care professional?

This paper explores these questions through both small-scale interviews (N=27) and a large-scale survey (N=303) that examine how various interface designs impact diverse users’ accuracy and confidence in interpreting the results of medical tests. In doing so, this paper makes several contributions:

1. A characterization of the advantages and challenges of using personal informatics technology to self-gather and interpret various types of medical data, including insights into situations where hesitation is warranted before deploying mobile health–based interventions
2. Definitions for measuring 2 specific problematic self-assessment scenarios, false confidence and false hesitance, along with our results regarding how various feedback formats and demographic attributes can predict these constructs
3. A set of concrete design recommendations to support users’ accuracy and confidence in interpreting feedback from mobile health tests
4. A general discussion of how future personal health systems can move in more tailored directions to support a greater harmony among specific interface components, user characteristics, and qualities of a monitored aspect of health

### Health Apps and (Self-)Tracking

In the United States, ownership of mobile technology is incredibly pervasive, with 90% of people owning cellphones and 64% of people owning smartphones specifically [2,3]. Globally, it is estimated that by 2020, 80% of adults will have a smartphone [4]. The extensive data-capture capabilities of these personal devices allow individuals to track, both manually and passively, a wide range of data that have traditionally been gathered in a clinical or laboratory setting. For instance, 7 in 10 US adults now track a health indicator (blood pressure, mood, weight, blood sugar, sleep, etc) for themselves or for a loved one [1]. The research community has documented such individuals’ “lived informatics” practices [5,6] and how they collect and use personal data to make changes in their lives [7]. However, an important and understudied consideration is how people are interpreting these self-gathered results, including the accuracy of their interpretations.

### Patient Interpretation of Medical Test Results

According to fuzzy trace theory, when making decisions, people rely on the “gist” of the information they receive, or their interpretation of the bottom-line meaning, instead of verbatim details, which explains why precise information is not necessarily effective in supporting medical decision making [8]. Previous work investigating the effects of patients being given direct access to their (clinician-gathered) personal health records has shown mixed results [9]. Specifically, although patients can feel an enhanced sense of control over their health, direct data access brings risks, including patients incorrectly interpreting the data or taking the wrong action in response.

Another concern of direct medical data access relates to issues of health literacy. A trained clinician can interpret test results with an implicit awareness of how values map onto severity or where thresholds for action lie—information that is unfamiliar or invisible to most patients [10]. Such challenges are compounded by the fact that in the United States, low numeracy is widespread, and written information about tests and their results are often provided at higher reading levels than many patients can manage [11] or in presentation formats that are perceived as uninformative [12]. Similarly, studies have found that many people experience difficulty in interpreting health information from graphs (ie, low graph literacy) [13,14]. Relatedly, diverse groups of people may respond differently to the same image-based feedback because of individual differences (eg, gender [15]) in visual perception, such as processing static versus animated images [16] or in the strength of reactions to pleasant or unpleasant imagery [17-19].

Furthermore, some groups of patients have highly variable relationships with health care as a whole. Racial and ethnic disparities in medical access and quality have been extensively documented [20], and some groups are more likely to experience bias and a lack of cultural understanding in health care [21]. Such problems could potentially translate into less involvement in the self-monitoring process to begin with or less confidence in interpreting health data. On the other hand, patients with higher levels of education may be more self-monitoring savvy and confident, given that research finds they are often better able to manage self-care regimens [22], are faster to adopt new medical technologies [23], and are more likely to use preventative care [24].

Altogether, such differences in comprehension and confidence between groups must be considered in the context of personal informatics and the interpretation of health data. Given the widespread acceptance of smartphones and self-tracking technologies, sophisticated personal medical tests will be a reality for the general population in the near future. The important implications of these tests require informed design of the interfaces used to present test results for general use. Although significant prior work has focused on individuals’ use of personal informatics tools [25,26], there is a lack of research that considers how various design strategies might impact users’ ability to interpret their own health measures outside of a clinical setting, along with their confidence in these interpretations.

## Methods

### Overview

This paper investigates individuals’ interpretation of health data outside of a clinical context. To do so, we used NutriPhone [27], our prototype system (see Multimedia Appendix 1) that transforms any mobile device into a point-of-care biomarker assessment tool by combining blood testing strips, a custom hardware accessory, image analysis software, and a user-facing app that delivers diagnostic reports.

### Preliminary Investigation

To gain qualitative insight into how people interpret medical data through NutriPhone, our preliminary investigation (Cornell Institutional Review Board Protocol ID#1410005065) used direct observation and dialogue in an interview-based lab study. We used an on-campus recruiting system to recruit participants (N=27, 20 female, aged 18-45 years). A total of 24 were undergraduate students who were compensated with course credit, and the remaining 3 were academic staff who volunteered their time. Interviews lasted approximately 10 min, were conducted in person, and were audio-recorded and transcribed.

**Figure 1:**
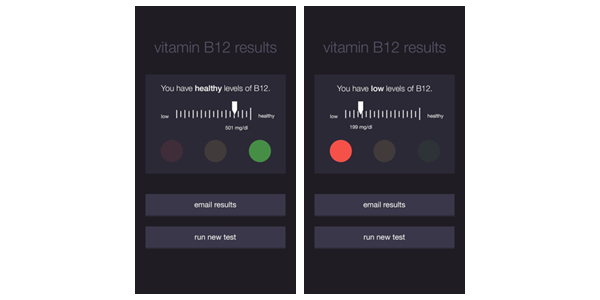
NutriPhone interfaces presented to participants in the preliminary investigation. Two variants of the interface showed a healthy result (left) and an unhealthy result (right). Both variants incorporated textual, graphical, numerical, and color design components.

Because the goal of the preliminary investigation was not to identify which design elements maximized interpretation accuracy but rather was aimed at observing and discussing participants’ process of interpretation and how various design choices impact it, we used a hybrid interface design. Specifically, we combined textual, graphical, numerical, and color components to reflect the predominant formats of visual feedback used by personal informatics systems and to appeal to multiple types of literacy [28]. For the health indicator, we chose to present vitamin B12 levels. Because vitamin B12 deficiency is fairly uncommon in developed nations, doctors rarely test vitamin B12 in isolation or discuss it with their patients [29], meaning our participants were unlikely to have prior knowledge about and would need to rely on our interface for interpreting the data. We implemented 2 versions of the interface, which can be seen in Figure 1: the “Healthy Result” (left) displayed a B12 level within the US National Institutes of Health–recommended reference range, and the “Low Result” (right) displayed a B12 level lower than this reference range [30].

To begin, participants were provided with a link to access the NutriPhone app on their personal smartphones, with the interface variant randomly assigned (14 and 13 participants saw the healthy and low variants, respectively). We told participants that the purpose of the app was to “help people run blood tests on their own without a health care practitioner,” and they were asked to imagine that they had already completed the testing procedure. We next asked participants to describe their test result and then followed up with questions about how they understood (or did not understand) their result, their usual method for interpreting medical test results, and overall impressions.

The results from the preliminary investigation pointed in 2 directions. First, 25 out of 27 participants (93%) correctly interpreted the test results (ie, correctly answered that their result was high or low when viewing with the high or low interface). However, despite their overall accuracy, 20 out of 27 participants (74%) also expressed confusion and doubted their interpretations. When asked what their test result meant, responses were often a variant of “I don’t know” or “I have no idea what that means.” Such doubt is important to consider, as it can inhibit the translation from insight to action, even if a person’s interpretation is in fact accurate.

Participants also wondered about the potential influence of individual or demographic characteristics on their result. Finally, several participants wanted to see an average or “typical” score or range to help them situate their results within the larger population, whereas a few wanted a more binary presentation that simply indicated whether their result was problematic or not.

Our preliminary findings left us with some unexpected outcomes and unresolved questions. In particular, we did not anticipate that such highly accurate interpretations would be accompanied by such confusion and uncertainty. Furthermore, we did not directly analyze which interface components supported correctness or contributed to doubt. Finally, having only looked at 1 biomarker, we were left wondering how participants would interpret other more well-known biomarkers with different reference ranges and whether those conclusions would be made more confidently.

### Main Study

To pursue these goals, our main study (Cornell Institutional Review Board Protocol ID#1410005065) focused on 3 different interface designs that present numerical, textual, and graph-based feedback:

1. *Number*: Biomarker level is presented as a number, providing only the raw measurement that would result from a blood test.
2. *Natural language*: Biomarker level is presented using natural language text that explains whether the biomarker level is considered low or high.
3. *Graph*: Biomarker level is presented graphically, with a marker at the measured value. The one-dimensional chart includes “low” or “high” anchors to provide orientation.

As mentioned earlier, these design styles were chosen to reflect the conventional feedback formats found in personal informatics systems and to appeal to distinct types of literacy [28]. Although informal pilot testing indicated that participants typically correctly interpreted green as healthy and red as unhealthy, we chose not to test a color-based feedback design because of inherent accessibility issues. Specifically, other cultures may ascribe these colors with different meanings [31], and the widely used “stoplight”-style color system for risk presentation [32] is indistinguishable for individuals with deuteranopia (insensitivity to green light, commonly known as red-green colorblindness).

Next, to broaden our variety of examined medical data, we focused on the following 3 biomarkers: vitamin B12, procalcitonin (PCT), and cholesterol. First, these biomarkers vary in terms of participants’ expected prior familiarity with them. Similar to B12, participants were unlikely to have prior knowledge of and know how to interpret PCT, which is used to diagnose bacteremia and septicemia [33]. In contrast, cholesterol is a more commonly known health marker, making participants more likely to be aware of what constitutes healthy levels. Our selected biomarkers also vary in terms of whether a higher or lower measure constitutes a healthier or an unhealthier result. As previously discussed, health consequences effectively only exist for low levels of vitamin B12. Conversely, PCT is problematic at high levels and has no medical consequences for very low or zero levels, and cholesterol similarly carries medical risk only at high levels.

**Figure 2:**
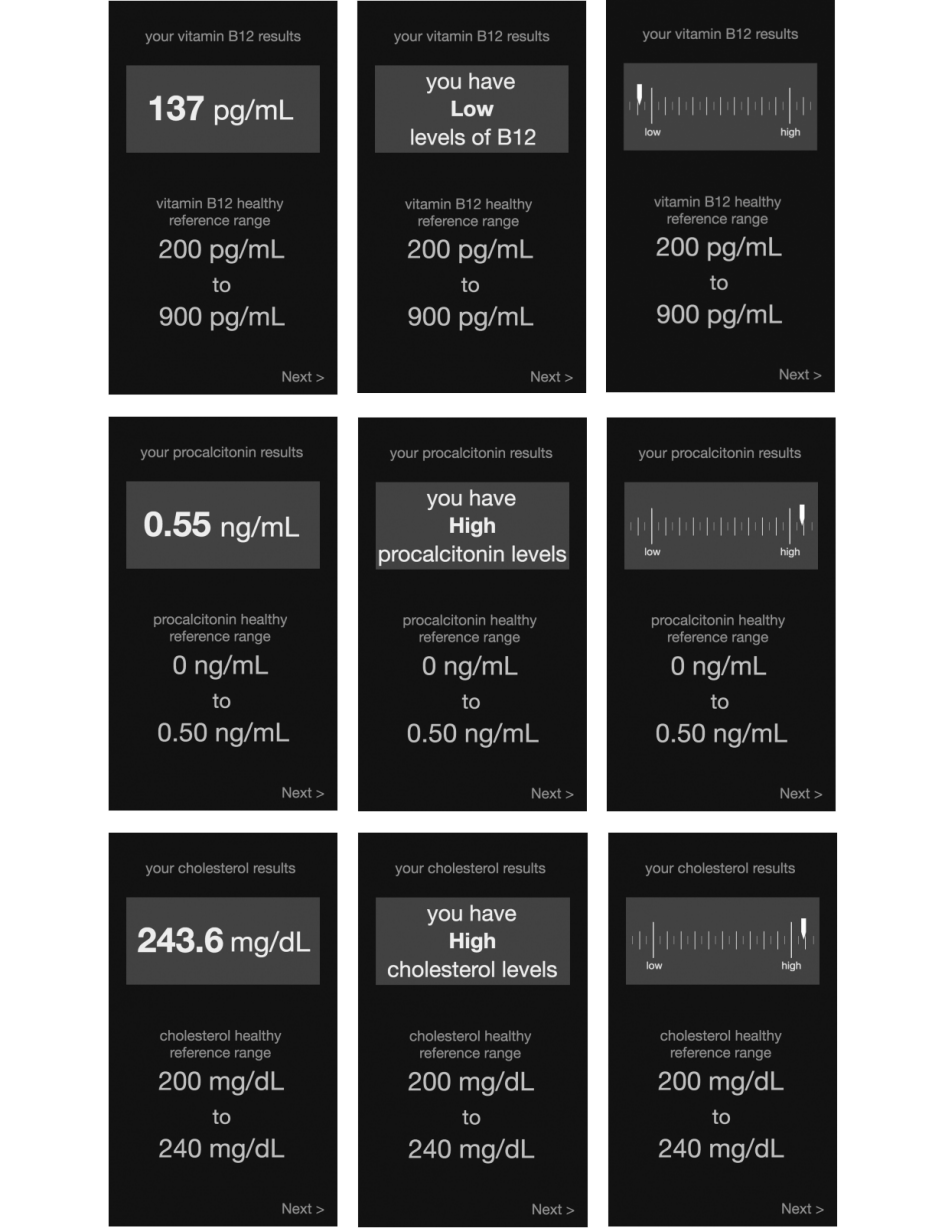
Our main study tested 3 feedback designs (number, natural language, and graph—left to right) with 3 biomarkers (vitamin B12, procalcitonin, and cholesterol—top to bottom).

For each of the 3 designs, we created mock-ups for each of the 3 biomarkers, resulting in 9 interface variants, as seen in Figure 2. Each variant included a “healthy reference range” for the respective biomarker at the bottom of the result screen. These reference ranges resemble what a patient would receive in a clinical setting, and participants’ comments from the preliminary investigation suggested that these ranges would facilitate interpretation. Because our preliminary findings showed no statistical difference between accurate interpretation of healthy or unhealthy results, we chose to display only unhealthy results.

### Participants

To examine individuals’ interpretation, confidence, and overall reaction to these various interfaces, we deployed a Web-based survey in September 2016 through Qualtrics, a system through which we enlisted 303 participants (155 female, 147 male, 1 bigender), who received various incentives (cash, airline miles, redeemable points, etc) for their participation. After providing Qualtrics with the survey questions and format along with the number and desired demographics of participants, they performed the process of carrying out the survey. We excluded 2 respondents from our analysis: 1 bigender respondent, both to prevent undue influence on the results and to prevent potential deanonymization, and 1 respondent who entered an age of 6 years, which we considered as a typing error considering Qualtrics only recruits adults. This left 301 participants for the main analysis.

Demographic screening criteria based on Pew’s omnibus Internet survey [34] were used to ensure a diverse, demographically representative sample of US Internet users. Ages ranged from 18 to 90 years (mean 45.96, median 45, SD 16.34). Of 301 respondents, 100 had a 4-year degree (33.2%), 67 had some college degree (22.3%), 48 had a high school degree (15.9%), and 45 had a professional degree (15.0%). Annual household incomes ranged from US $40,000 to more than US $200,000 (mean US $88,210, median US $80,000, SD US $3142). Racially, 201 out of 301 respondents identified as white, 66.8%); 59 identified as Hispanic, Latino, or Spanish origin (19.6%); 37 identified as black, African American, or Negro (12.3%); and 12 identified as Asian Indian, Chinese, Filipino, Japanese, Korean, Vietnamese, or other Asian (4.0%). Racial categories were *not* mutually exclusive; individuals who identify as multiracial were allowed to select multiple races.

### Procedure

Participants first gave informed consent after reading about the purpose, time commitment, question types, risks and benefits, confidentiality, data storage, and principal investigator for the study (see Multimedia Appendix 2). Next, participants were given background information about NutriPhone and then randomly assigned to 1 of the 3 biomarkers (B12, PCT, or cholesterol). Adapting materials from Mayo Clinic [35] and Medline Plus [21,36], we then gave participants some background about that biomarker, including medical consequences of and how to counteract unhealthy levels.

Next, they were shown an unhealthy result via 1 of the 3 interface designs (number, natural language, or graph) and asked a series of questions, starting with “My levels of [biomarker] are...” with choices of “too low,” “healthy,” “too high,” and “unsure.” We also asked participants about how confident they were in this interpretation. Participants were next asked a series of multiple-choice questions about how they might use NutriPhone, free-response questions about their general impressions of the system, and demographic questions. A few attention check questions (eg, “What planet are humans from?”) were deployed throughout the survey, and all participants correctly responded to these checks. Respondents were not able to change their responses after they had been submitted.

## Results

### Operationalizing and Predicting Problematic Interpretations

In undertaking our quantitative analysis of survey responses, we identified 2 problematic scenarios. We term the first scenario “false confidence,” which represents a respondent having above-average confidence despite inaccurately interpreting a result.

This situation is particularly concerning, given that it equates to a person incorrectly believing he or she is healthy when that is not the case. Second, we observed instances of what we call “false hesitance,” where participants accurately interpreted the result they saw but had below-average confidence. Although potentially less health hazardous than false confidence, such situations emerged as a consistent theme in our preliminary investigation and could still lead to hesitation or failure to take an appropriate course of action to address an unhealthy result.

To operationalize false confidence, we created a binary variable capturing both whether a respondent supplied an incorrect interpretation of the result and whether his or her confidence was above the mean confidence of all respondents who supplied incorrect interpretations. This approach labeled 33 out of 301 respondents (10.7%) with false confidence. Operationalizing false hesitance followed a similar procedure in which we identified respondents who interpreted the result correctly but with confidence below the mean confidence of other correct respondents. This approach labeled 66 out of 301 respondents (21.9%) with false hesitance.

To determine the factors most strongly associated with false confidence and with false hesitance, we constructed 2 binary logistic regression models, 1 for each outcome, using all subsets model selection. Potential predictors included experimental condition (ie, the design variant and the health condition the respondent saw), age, gender, education level, household income, and 1 binary variable each for the racial categories of Asian, black, Hispanic, and white, as well as interactions between the design variant and each of the other potential predictors. Model selection for false hesitance failed to converge on a significant model. That is, no subset of the variables we collected significantly predicted which participants would correctly interpret the interface and yet have low confidence in their answer.

We therefore focus on false confidence, for which model selection resulted in a model with a *P* value of .004, an area under the curve score of 0.77, and a McFadden pseudo *R*^2^ of .15, all of which indicate a good fit. Table 1 presents the model’s details. Results are presented in terms of odds ratios; an odds ratio of +2.0 means that a 1-unit increase in that predictor equates to a participant being 2 times *more* likely to exhibit false confidence, whereas an odds ratio of −2.0 means that a 1-unit increase in that predictor equates to a participant being 2 times *less* likely to exhibit false confidence.

As Table 1 shows, we found several main effects and a few interaction effects, with the strongest significant effects relating to design, gender, race, and education. Specifically, participants who saw the natural language design were the least likely to exhibit false confidence by far, and women who saw the graph design were over 8 times less likely to have false confidence. The lack of false confidence shown for the natural language design makes sense given the fact that participants were screened for English-language proficiency, especially when compared with the graph and number designs, for which we would not expect to see false confidence given the widespread low graphical and numerical literacy in the United States [13,14].

**Table 1:** Model for false confidence, showing odds ratios and *P* values. Main effects occur for the natural language design and for race. Interactions occur between the graph design and gender, the natural language design and race, and the natural language design and education.

Predictor		Odds ratio	*P* value
Graph design		+2.29	.56
Natural language design		−421.47	.02
Female		+1.06	.93
**Race**			
	Asian	+25.30	.02
	Black	+7.38	.19
	Hispanic	+6.19	.04
	White	+13.99	.01
Education		−1.14	.53
Graph design × female		−8.67	.04
Natural language design × female		−3.76	.27
Graph design × education		−1.07	.83
Natural language design × education		+3.06	.02

On the other hand, false confidence was more likely among participants who self-identified as Asian, white, and Hispanic. Finally, with the natural language design, participants who were more educated were, for each 1-unit increase in education level, approximately 3 times more likely to have false confidence. The observed findings regarding false confidence may reflect the findings of the National Assessment of Adult Literacy [37], which showed that white and Asian/Pacific Islander adults had higher average health literacy than adults of other races and that average health literacy increased with each higher level of educational attainment. However, our work is also somewhat at odds with these findings, as these groups were falsely confident in incorrect interpretations of health data, suggesting the possibility that groups with relatively higher health literacy could be more prone to unknowingly misinterpreting health data.

### Promises and Risks of Interpreting Self-Gathered Medical Data

Encouragingly, 242 out of 328 participants (73.8%) interpreted their result accurately, in spite of the typical lack of common knowledge about vitamin B12 and PCT described earlier as well as the aforementioned low numeracy and graphical literacy rates [28,38]. These findings demonstrate the possibility of understandably conveying health information (even about less familiar health indicators) through mobile interfaces, as long as the design of that feedback provides enough context.

We saw additional glimpses into the potential of giving people access to their medical data as a number of respondents described a desire to use our prototype system to monitor their personal health. For example, participants expressed that the tool would be helpful for self-screening or could help ease nerves surrounding a health condition of personal concern. One participant told us how she could “save money from having to go to the lab to have my blood tested; I could do this all in my own home.” Other respondents envisioned using the system as a way to get actionable guidance when making health-related lifestyle changes and were open to the system additionally providing more prescriptive behavioral feedback, with 1 participant expressing that it would “...help control their health and be on top of things.”

At the same time, our results also highlight disadvantages of allowing individuals to interpret their own medical data and areas where personal informatics may be less appropriate. Although the majority of our participants correctly interpreted their results, many expressed confusion and questioned their interpretations: 20 out of 27 (74%) participants in the preliminary investigation expressed self-doubt, and 115 out of 301 respondents (38.2%) expressed low confidence in the main study. We believe that our addition of a “healthy reference range” with the result is largely responsible for this decrease in confusion between our preliminary and main studies. It is also possible that the main study’s survey-based methodology was more susceptible to social desirability bias compared with the more personal nature of the in-lab study, which may have encouraged participants to open up about interpretive insecurities.

Taken together with the fact that 84 out of 301 of main study responses (28.0%) were inaccurate, we observed that half of all study participants either inaccurately interpreted their result, lacked confidence in their interpretation, or both. Participant comments helped to shed light on the observed lack of confidence, with many expressing similar desires to “discuss [the results] with a doctor.” Other participants discussed self-doubt in result interpretation stemming from inability to correctly perform the test, with 1 person describing how “...there could be a lot of wrong readings if tests are not done properly,” and another saying how they “...would need a lot of information to be able to use it correctly and safely.” This hesitation and confusion presents a clear problem for providing people with the ability to collect and interpret health data, especially as systems such as NutriPhone are able to analyze increasingly complex and meaningful biomarkers. If people are unsure of their results, it undermines the aforementioned benefits of personal informatics tools, as “data that are not understood will always remain data unused” [10]. The potential for confusion among patients also sheds light on physicians’ mixed attitudes about whether patient access to medical data is a good idea, especially for abnormal results or for tests with vital consequences [39]. Regardless, as self-tracking gains increasingly mainstream popularity, direct access to medical data is becoming a reality, making investigations into effective ways to communicate mobile health data imperative to the future of personal informatics.

## Discussion

### Principal Findings

The results of our study provide a mix of implications regarding whether or not (and if so, how) personal informatics tools should support individuals in gathering and interpreting their own medical data. Overall, we find that a thorough understanding of the target audience is necessary before deploying any personal informatics tool and, especially for tests with vital consequences, suggest mobile health systems as a mediator between clinician and patient.

### Design Constraints and Recommendations

Overall, our findings suggest several design strategies for presenting mobile health data to maximize users’ ability to correctly and confidently understand them. Primarily, there is value in using a hybrid feedback design that includes multiple representational modalities (eg, numbers, words, visual graphics), as such a design allows a designer to tap into different literacies to increase a display’s effectiveness. We saw more accurate interpretation of results in our preliminary investigation, where we used a hybrid design, than in the main study, where participants viewed designs with only a single representational format. In cases where it is not possible to include multiple types of feedback in the interface (eg, mobile apps where screen space is limited or when presenting results from multiple tests simultaneously), we recommend ensuring that a design integrates text-based feedback, where natural language is used to convey whether a result is “healthy,” “high,” or “low.” The natural language design was least likely to cause false confidence among our participants, and among all of our tested design variants, the natural language and hybrid designs were interpreted correctly most often.

Next, we recommend including a “healthy reference range” along with any results given, especially for biomarkers or other health indicators with which a user is expected to have less preexisting knowledge. Participants’ confusion with the lack of a reference range in the preliminary investigation seemed to be alleviated once it was included in the main study.

Finally, it seems worthwhile to allow users to input personal details. Many of our participants expressed uncertainty about how such factors might influence their test results, which would in turn contribute to their lack of confidence in both their results’ reliability as well as their own assessment. In our main study, we captured and analyzed demographic variables such as age and gender, but participants also indicated their receptivity to supplying other personal data that can influence a given health condition (eg, a cholesterol diagnostic tool requesting weight information). Even for tests where these variables are not in fact relevant, such as for vitamin B12, the ability to input this information may alleviate the user concerns we observed and in turn increase their trust and acceptance of the system. Furthermore, our findings about how individual differences can impact interpretation outcomes suggest that there is an opportunity to dynamically adjust an interface’s feedback format to use the representation least likely to cause confusion or misinterpretation for a given person.

### Implications for Personal Health Informatics

The level of confusion and inaccurate interpretation observed in our investigation suggests situations in which personal informatics may be inappropriate. Our studies tested 3 health conditions and found that although the majority of participants could correctly interpret the data, their analysis was consistently couched in confusion. We also saw that different groups of people vary in their interpretation confidence and accuracy. These findings indicate that before a mobile health system is introduced, developers should first ensure that the biomarker being tested is one that users are comfortable self-tracking and produces results that people confidently understand how to appropriately act on. The ramifications of some users inevitably interpreting results incorrectly must also be considered, with situations in which a serious health issue goes untreated (ie, false confidence or inaccurate interpretation) being the most problematic.

With less well-known biomarkers or for populations who are more susceptible to making misinterpretations, we recommend using systems such as NutriPhone as a mediator between patients and health care providers. For example, a user could complete a routine blood screening using such a tool in the hours before an appointment with their clinician. Immediately after the test, the results are available for the patient, but the results are also sent to the clinician, who would discuss them during the appointment, including an interpretation of any abnormal findings and agreeing on a treatment plan together with the patient. If follow-up tests are appropriate, use of the tool could be continued for at-home monitoring. This scenario preserves many of the promises of personal health tracking while mitigating the potential risks our study identified. Patients would be able to perform ecologically valid self-tracking, interact directly with their medical data, and become empowered with a more active role and informed voice in their treatment. In addition, oversight by a health care practitioner would ensure appropriateness of follow-up actions, reduction of patient confusion, and avoidance of the aforementioned dangerous scenarios. Leveraging personal informatics technologies to transfer this type of health care management more directly into the hands of patients is attractive from an institutional perspective (eg, appealing to clinicians and insurance companies), especially in light of anticipated physician shortages in the United States [40], and our study indicates that patients themselves are receptive on a personal level as well.

### Limitations and Future Work

Finally, we would like to point out potential limitations of our research and lay out room for future work. First, the results we presented to participants were pregenerated data, not actual outcomes. Displaying mock data is a common practice in system evaluation and still enabled us to gain insights into our key research questions regarding how people interpret medical data using a mobile health system outside of a clinical setting. Using mock data also imposed much less burden and privacy risk for participants, as they did not need to collect and share potentially sensitive health information. That being said, participants may react differently if interpreting real diagnostics about their actual health, especially considering personal medical data have been shown to carry strong emotional connotations [21], which we did not observe in this study. A natural future step is therefore to explore individuals’ interpretation of their own diagnostic results presented through a mobile health system.

Next, although the design elements we tested (numbers, graphs, and words) demonstrated significant differences, this study represents a partial exploration of a vast design space. Future work would do well to consider other elements that might appeal to different kinds of literacies [12,14,28,38] (eg, other types of visual charts or perhaps entirely different interaction modalities such as audio- or tactile-based feedback). Similarly, it would be desirable to expand investigations into additional types of medical results. For instance, data such as body mass index (BMI) could be especially valuable, given that for measures such as BMI, knowing simply whether or not one’s value is “within normal limits” may not be sufficient.

Finally, this study captured participants’ interpretations at one point in time. Previous research [41] has found that some patients feel that long-term self-tracking is “effortful and time-consuming” and sometimes give up the practice out of frustration. Future work would benefit from considering potential learning or habituation effects and emotions arising from viewing subsequent tests over an extended period of time, especially considering this would be a typical experience for an individual managing a chronic condition.

### Conclusions

Medical technology is changing rapidly, with numerous devices and systems placing health information directly in the hands of patients. Personal mobile health tools that present feedback using formats similar to those we have examined in this research will likely become a similarly substantial part of medical care. With that day fast approaching, researchers and practitioners must be prepared to design effective tools that are not only comprehensible but also allow patients to be correct and confident in their interpretations and follow-up actions. For example, we find that user understanding is cultivated by natural language–based feedback as well as hybrid designs that integrate multiple different representational formats. Such design strategies and the broader implications identified by studies such as ours are key to ensuring that future generations of systems are appropriate and useable in nonclinical settings.

## Appendix

Multimedia Appendix 1 The NutriPhone system for point-of-care diagnosis and health monitoring consists of 3 parts: (1) a disposable test strip for blood sample analysis, (2) a portable optical reader that images the test strip, and (3) a platform-agnostic software app to process the images and provide a diagnostic result to the end user.

Multimedia Appendix 2 The preface text shown to participants gives background information about NutriPhone and, depending on the condition, background about a biomarker adapted from Mayo Clinic and Medline Plus.

## Abbreviations

BMI: body mass index
PCT: procalcitonin
