# Supplementary figures and images for: Supporting Accurate Interpretation of Self-Administered Medical Test Results for Mobile Health: Assessment of Design, Demographics, and Health Condition
Source: JMIR Hum Factors. 2018 Feb 28;5(1):e9. doi: 10.2196/humanfactors.8620 (PMC5856924; doi:10.2196/humanfactors.8620)

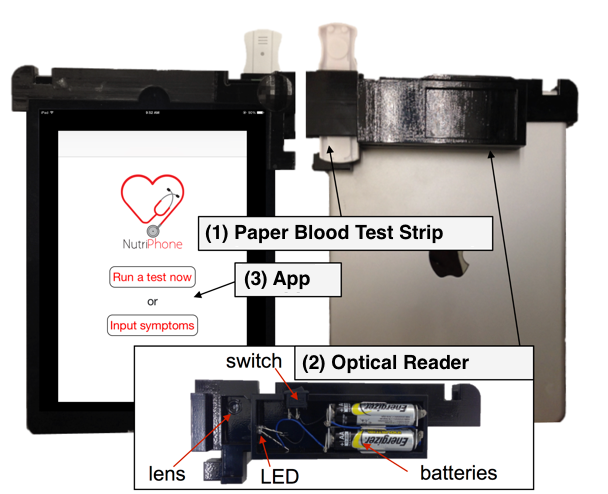

Supplement: Multimedia Appendix 1 [file humanfactors_v5i1e9_app1.png]
