# Supplementary material for: Supporting Accurate Interpretation of Self-Administered Medical Test Results for Mobile Health: Assessment of Design, Demographics, and Health Condition
Source: JMIR Hum Factors. 2018 Feb 28;5(1):e9. doi: 10.2196/humanfactors.8620 (PMC5856924; doi:10.2196/humanfactors.8620)
Supplement: Multimedia Appendix 2 [file humanfactors_v5i1e9_app2.pdf]

Preface shown to all participants:

What is Nutriphone?

Many health conditions can be monitored using blood tests. However, blood tests generally need to be administered by medical technicians and sent to a lab for processing, which takes time. Test results are also typically sent to a doctor's office rather than directly to patients.

To address these issues, we have developed the Nutriphone, a smartphone-based system for at-home blood work and nutrition monitoring. The Nutriphone system consists of three parts: (1) a small, plastic accessory which clips over a smartphone or tablet camera, (2) a custom paper test strip, and (3) a smartphone or tablet app to guide the user and process the test results.

To use the device to perform your own blood test, you start up the Nutriphone app and follow step-by-step instructions. The app will direct you to prick your own finger with a sterile lancet and collect a single droplet of blood. You then place the blood droplet onto the supplied test strip, and insert the test strip into the hardware accessory. The following graphic shows the device as well as a brief pictorial representation of what a user needs to do.

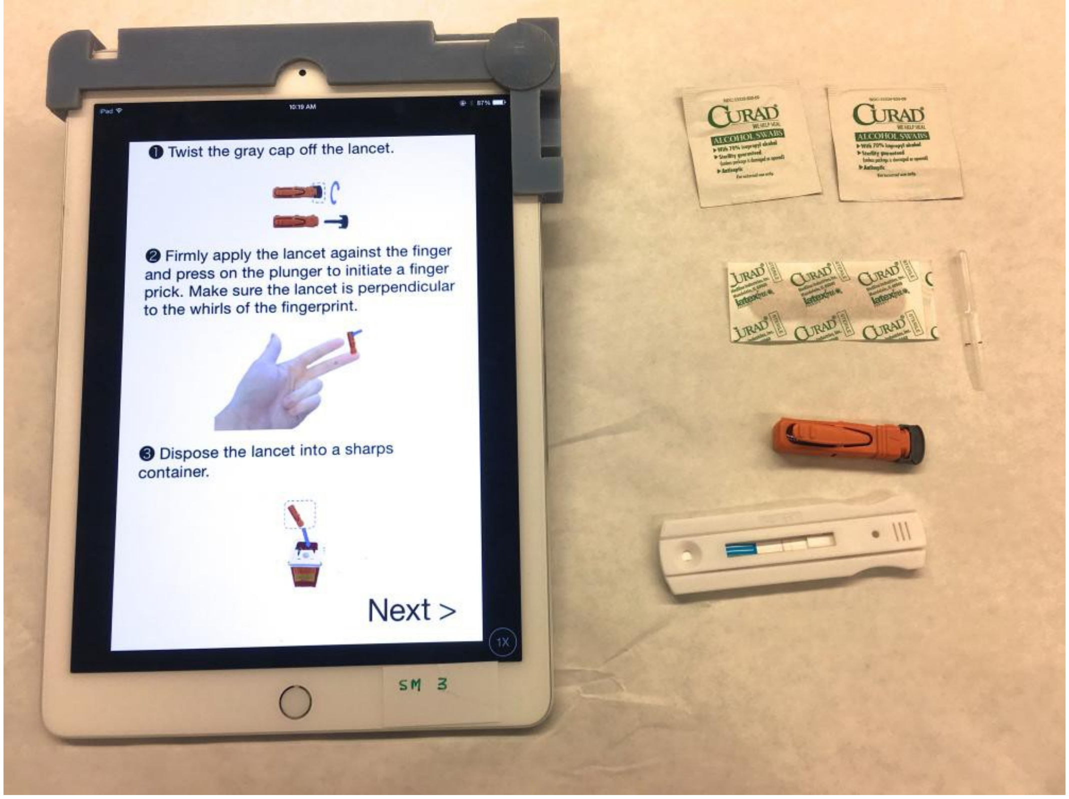

The Nutriphone app will then take a picture of the test strip, process the image to

determine your nutrient status, and display the results on the screen. The whole process from starting the app to receiving test results takes around 10-15 minutes.

Information shown to participants in Condition 1: Vitamin B12

A version of NutriPhone has been developed to test for vitamin B12.

Vitamin B12 is a nutrient that helps keep the body's blood and nerve cells healthy, as well as being involved in making DNA. B12 deficiency is associated with tiredness, weakness, loss of appetite, weight-loss, anemia, nerve damage, and behavioral changes such as memory loss or depression.
B12 is not known to cause any harm, and there is not a known "overdose" level.

Treating a vitamin B12 deficiency usually requires taking a vitamin supplement.

Information shown to participants in Condition 2: Procalcitonin

A version of NutriPhone has been developed to test for procalcitonin.

Procalcitonin is a protein associated with the immune system. Elevated levels of procalcitonin often indicate a bacterial infection, also called sepsis. They can also occur with inflammation, such as from major burns, severe trauma, or major surgery. There is not a known "deficient" level of procalcitonin.

Treating high procalcitonin levels usually requires taking a prescription antibiotic.

Information shown to participants in Condition 3: Cholesterol

A version of NutriPhone has been developed to test for cholesterol.

Cholesterol is a soft, wax-like substance found in all parts of the body. Your body needs a little bit of cholesterol to work properly, especially your circulatory system. However, too much cholesterol can clog your arteries and lead to heart disease.

Treating unhealthy cholesterol levels can require major lifestyle changes, such as in diet or physical activity, taking prescription medication, or potentially even surgery.
